# Evaluation of an Online Gottman’s Psychoeducational Intervention to Improve Marital Communication among Iranian Couples

**DOI:** 10.3390/ijerph18178945

**Published:** 2021-08-25

**Authors:** Neda Deylami, Siti Aishah Hassan, Naser Abdulhafeeth Alareqe, Zaida Nor Zainudin

**Affiliations:** 1 Department of Human Development and Family Studies, Faculty of Human Ecology, University Putra Malaysia (UPM), Serdang 43400, Malaysia; nedadeylamii@gmail.com; 2 Department of Counselor Education and Counseling Psychology, Faculty of Educational Studies, Universiti Putra Malaysia (UPM), Serdang 43400, Malaysia; zaidanor@upm.edu.my; 3 Department of Educational Psychology and Counseling, Taiz University (TU), Taiz 6803, Yemen; nalareqe@yahoo.com

**Keywords:** marital communication, online Gottman’s psychoeducational intervention, constructive communication, withdraw–demand communication, mutual avoidance communication, Iranian couples

## Abstract

Amounting evidence indicates that insufficient knowledge of marital communication skills leads to destructive interactions and poor marital adjustments in couples, especially during stressful situations. Despite the high effectiveness of Gottman’s psychoeducational intervention, there is a lack of study on the online Gottman’s psychoeducation intervention (O-GPI) to improve marital communication and dyadic adjustments. The aim of this study was to evaluate the effectiveness of O-GPI on the improvement of marital communication patterns among Iranian couples. Method: The study followed a single-blind parallel group in a randomized controlled trial using an experimental longitudinal design, comprising 72 heterosexual couples living in Shiraz, Iran, with a 1–7-year marital age and no severe marital problems. The experimental group received eight consecutive O-GPIs via the Zoom platform, while the control group received information related to parenting skills via email. The outcome measures were the three patterns of communication: (i) constructive communication; (ii) demand–withdraw communication; and (iii) mutual avoidance communication—the screening measure was the dyadic adjustment scale. Results: The findings indicated that O-GPI could improve couples’ constructive communication significantly (45% for husbands and 40% wives) and decrease their total demand–withdrawal (51% for husbands and 65% wives) and mutual avoidance communication (60% for husbands and 62% wives). Limitations: Due to the homogenous nature of the sample, generalizations should be made with caution. Conclusions: This study demonstrates the feasibility and effectiveness of the online Gottman’s psychoeducational intervention to improve couples’ communication patterns.

## 1. Introduction

### 1.1. Study Background

Generally, human wellbeing improves through developing and maintaining romantic relationships as an important part in all transitional stages of human life, from dating in adolescence, cohabitating in emerging adulthood, and ultimately marriage in adulthood [1]. Marriage has been addressed as one of the leading sources of both support and stress for adults [2,3]. In successful marriages, couples experience lower psychological distress and higher wellbeing [4]. In turn, insufficient marital-life knowledge of constructive interactions between couples can lead to an unsuccessful marriage, dissolution and divorce [5,6], as well as generate mutual avoidance and demand–withdraw patterns, which are destructive communication patterns that can destroy marriages [7,8].

Healthy interactions between members of a family, particularly couples, is negatively influenced by out-of-control issues, such as financial problems [9], parenting issues, work commitments [10], personal difficult behaviors, mental health domains [11] and transformation in the family lifecycle stages [12]. On the other hand, intimate interactions between couples can generate the comfort, security and support that are essential for couples to cope with stress. Therefore, handling the effects of external and internal stress is a very critical problem to enhance how partners interact with each other. Couples experience salient challenges in their marital life. Without addressing these challenges, it will make them more vulnerable to destructive interactions, with direct consequences to their own and their partners’ mental and physical health [13]. Couples who face more stress are more likely to have a less satisfied relationship and, in turn, are more at risk of destructive communication, dissolution and finally divorce [14]. Therefore, the especially critical challenge in marital life is maintaining an effective intimate relationship by achieving the appropriate knowledge of marital life to improve couples’ physical and mental health levels.

Divorce rates are dramatically increasing around the globe. In 2020, the highest divorce rate was reported in Russia (4.8 divorces per every 1000 residents), while the USA and Iran reported 2.5 and 2.3 divorces per every 1000 residents, respectively [15]. Statistical reports have shown that, globally, 50% of first marriages end in dissolution and divorce [16]. Additionally, in the Iranian context, Askari [17] reported from the National Youth Organization that more than 273 divorce cases occur every day and the divorce rate has skyrocketed by 80% since 2010. This is because Iranian families in recent decades have been influenced by an uncertain range of socio-economic, cultural and religious factors, such as modernization, the Islamic revolution, a destructive eight-year war between Iran and Iraq and economic problems [18]. Besides these factors, an increase in the marriage age is another social change that has affected Iranian families. According to a report from the National Organization for Civil Registration in Iran under the Ministry of Interior [19], from 2005 to 2014, the percentage of unmarried men older than 35 had risen from 6.7% to 10.2%. In the same period, the percentage of unmarried women over 30 had increased from 6.3% to 13.8%. The accustomed marriage age ranges were 20–34 for men and 15–29 for women. It also has been reported that there was a 7.2% decrease in marriages and 8.2% increase in divorces in 2014.

Other social changes in Iran are the decrease in family sizes, an increase in the rate of select-marriage and the selection of spouses among young people. There is also a rise in polygamy, women’s rights to file for divorce, a surge in women’s participation in social activities, higher quality of education and availability of modern jobs. Accordingly, couples have experienced more marital dissolution, unhappy marriages and emotional disengagement [20]. Researchers have suggested that one of the main effective factors of marital dissolutions is the lack of dyadic competencies, such as communication skills, conflict management and emotional disengagement in both partners [21,22,23]. It is important to note that often the most preventive attempts for dissolution and divorce are education, counseling or family therapy. Recent developments in family counseling have heightened the necessity of couples’ training and family education programs instead of family therapy [24,25].

A healthy long-lasting marriage will be achieved if couples attend family education training programs, learn skills and do their best accordingly together to improve their communication skills [26,27]. Despite the numerous successful research studies on couple psychoeducational intervention for marital quality, divorces and marital dissolutions are still rising due to the deficiency in marital skills, especially marital communication skills [26,27]. Consequently, couples with the relevant marital knowledge can regulate affection, manage tension and achieve health and happiness more easily than couples that are in discordant marriages [18,28,29]. The works of Moshtaghi [30] and Wallerstein [31] indicate that learning marital skills, such as information about constructive–destructive marital communication patterns, is essential to ensure a successful marriage. The acquired information and knowledge can be used to evaluate how a couple creates and maintains marital satisfaction by attending counseling sessions or couple training programs (such as Gottman’s psychoeducational intervention).

The studies on different couple interventions have shown that the intervention can improve marital satisfaction among unsatisfied couples in terms of decreasing demand–withdrawal communication patterns [2,24,32,33,34,35,36,37,38]. However, Iranian couples have a low attendance rate in family and couple therapy sessions. This is due to them, among others, believing that marital problems are a private and personal issue, that marital issues should not be discussed outside of the family, confiding in older male relatives, family economic problems or a lack of confidence in the ability of the counselor [39]. Therefore, training can be a good alternative for families and have a fraction of the cost of couple therapy.

The current study adopts a large body of theory and research on the effects of Gottman’s approach to improve marital interaction, particularly when they are under the stress of challenging events without the necessary face-to-face counseling sessions. This study attempted to conduct online psychoeducational intervention for married couples, particularly using video conferencing, because of its many advantages when compared to face-to-face sessions. These advantages include decreased time consumption and transportation costs, selection of a comfortable place, more confidentiality, and a decreased sense of stigma for the participants [40,41]. Based on previous studies, couples who acquired skills from Gottman’s psychoeducational intervention, which is one of the top ten effective family approaches, can increase their marital satisfaction and prevent marital dissolution [6,24,42,43,44,45,46]. It should be noted that virtual intervention could foster married life satisfaction, which is required to help couples in giving and receiving support, communicating, conflict managing and problem solving [14]. These skills provide a strong foundation and structure for every family.

### 1.2. Problem Statement

Despite numerous research studies having been conducted on the Gottman intervention, with the accumulated evidence clearly showing the positive effects of the Gottman method on marital communication [2,32,45,46,47,48], far too little attention has been paid to empirical evaluation of the online Gottman’s psychoeducational intervention, particularly in Iran. Some inconsistencies exist in the literature. For example, Iranian research to date has tended to evaluate Gottman’s approach in individual counseling sessions rather than couple’s training. Additionally, on the other hand, Iranian couples are rarely inclined toward couple therapy [17,18,24,36,39,44,47,49,50,51,52,53,54,55]. Indeed, among Iranians, training is more preferable compared with family therapy or counseling [53,54]. “Training” is a preferable alternative for them. Furthermore, the rate of attendance in training sessions among male participants is not very impressive, and they are less likely to seek professional help [56]. Similarly, Iranian husbands also have low attendance in family training programs and a negative attitude towards professional help. When they are supposed to attend, they prefer to do so alone. 

Therefore, this study aims to experimentally evaluate the effects of the online Gottman’s psychoeducational intervention and encourage both Iranian husbands and wives to attend it to improve their marital communication patterns. It was predicted that this highly effective intervention could increase constructive communication patterns and decrease destructive communication patterns among couples when they face any uncertainty challenges. It was interesting to determine whether the interactions of time, group and gender can change the mean scores of marital communications compared with the control group. Additionally, this change was tested again almost one year after the intervention.

### 1.3. Summary of the Theories and Literature

#### 1.3.1. Family Communication Theory

Family communication theory, as one of the early theories of communication in a family, is a subset of the relationship theories introduced by Fitzpatrick [57]. This theory discusses the relevant issues surrounding family communication and from there developed a general model of the role of relational schemas for interpersonal communication. The family communication theory identified four family types, which are consensual, pluralistic, protective and laissez-faire, based on the dimensions of the conversation (the degree to which members can talk freely and spontaneously) and conformity orientation (the degree to which family members encourage or discourage uniformity of beliefs and attitudes, as well as behavioral regularity) [58]. Based on this theory, the concept of marital communication patterns was described as below:

The Patterns of Marital Communication

Marital communication patterns can be categorized as dysfunctional or functional. Communication is a reliable differentiating criterion between distressed or dysfunctional and non-distressed or functional couples [59]. While functional interaction patterns as a mutually constructive pattern bring about satisfying consequences in a marriage, dysfunctional interactions, such as demand–withdraw and mutual avoidance patterns, provide misery, dissatisfaction and, eventually, dissolution. Couples with dysfunctional communication cannot be satisfied with their relationship [60,61]. Hence, they will show negative communication instead of positive communication. These couples have many conflicts without any solution, and the unresolved problems affect their mental and physical health [62,63].

Mutual Constructive Communication Pattern

Mutual constructive communication patterns are positive and functional. Couples showing this pattern respond with warmth and friendliness to their partners. They are interested in interaction and show affection, manage conflict constructively and share humor. One important indicator of constructive communication is validation. Validation is the acceptance of a partner’s point of view, which leads to respect in the interaction [64]. Couples that are validated usually try to maintain eye contact and increase their understanding of each other by paraphrasing and asking questions in the right situations, even in disagreement. A couple with a constructive communication pattern has mutual discussions, and both parties of the dyad try to negotiate and discuss problems in a calm position and accept the other’s point of view. Both members of the dyad express their feelings to each other and describe their problems, needs and individual expectations. They suggest possible solutions and negotiate with each other. They have more positive interactions than negative communication between each other as a dyad [26]. Even if these couples have a negative interaction, it is natural, and these behaviors are irrelevant to disasters in the relationship [5]. Gottman suggested that a five-to-one ratio of positive-to-negative feelings during conflict situations characterizes a constructive communication pattern [65]. These couples can keep calm and de-escalate their physiological arousal [29]. They are used to repairing their interaction if it does become negative. Constructive communication encourages a couple to move toward compromise. These couples avoid ailing and failing situations because they prevent escalation of negative effects and emotional disengagement. These “master couples” want to stay together and they are satisfied with their relationship [5,65,66].

Demand–Withdraw Communication Pattern

Another pattern of marital communication is the demand–withdraw interaction, where one spouse makes demands and the other withdraws. This pattern represents the amount and intensity of closeness and intimacy between each spouse [67]. Couples with a demand pattern achieve more intimacy and interaction, and desire more closeness. Generally, they are in a “one down” position. The person who has demand forces the partner to communicate and tries to do so through emotional requests, criticism and complaints. The withdrawn partner wants to be dominant in the relationship, to be more independent and autonomous, and to avoid interaction in general. The withdrawn partner has manners such as defensiveness, passive inaction or “stonewalling” [66,68].

In the demand–withdrawal pattern, a wife or husband makes demands, solicits for change and wants to communicate or criticize, while the other is defensive and avoids inaction. A number of studies have described that the demand–withdrawal pattern frequently happens among dissatisfied couples than satisfied couples. Additionally, the pattern of wives demanding and husbands withdrawing is more widespread than wives withdrawing and husbands demanding [7,67,69]. This pattern is one of the negative communication patterns that is expected to decrease after couple intervention [18,52,70].

It has been shown conclusively that the withdrawn partner is in the position of power and wants to stabilize the distance, followed with polarized roles [71], and withholds resources that the demanding partner wants. Meanwhile, the partner in the demand position is unable to achieve the desired changes without cooperation from the withdrawn partner [7,8,63,72]. These demands can range from more intimacy to more cooperation with household chores. Spouses with a withdrawing pattern usually avoid discussion by shifting subjects, remaining silent or even removing themselves physically from the conversation.

This communication pattern is significantly associated with dissatisfaction in the relationship, intimate partner violence [68,73,74,75] and relationship dissolution [76,77]. Additionally, there is evidence that wives utilize the pattern of demand–withdraw interaction by presenting nagging, negative affect and criticism within conflict, while husbands avoid conflict or discussion. This pattern is more common between distressed couples than happy couples.

Mutual Avoidance Communication

In the mutual avoidance communication pattern, both spouses avoid interacting with each other. Both of them try to abstain from both positive and negative interaction. They are also trapped in stressful situations and experience negative feelings. However, they are unable to downregulate the negative effect. A longitudinal study showed that these couples avoid each other to maintain calm for themselves and their partners [5].

If a couple learns how to communicate in a positive manner through Gottman’s principles, they will be in a better position to find more satisfaction and create more stability in their lives. Positive communication is functional interaction. Eight factors (combined into seven items) can affect communication [5,78]. These factors are typically associated with functional or dysfunctional communication and can predict dissolution or long-lasting marriages [37,79] and are listed below:

1. More negativity than positivity;
2. Escalation of negative effect;
3. Emotional disengagement and withdrawal;
4. The failure of repair attempts;
5. Negative sentiment override (NSO);
6. Maintaining vigilance and physiological arousal;
7. Solvable problems and perpetual issues.

#### 1.3.2. Gottman Couple Theory

The Gottman couple theory (1999) identified by John Gottman is an evidence-based approach in couples therapy that encourages therapists to assist couples to acquire a deep sense of understanding, awareness, empathy and connectedness within their relationships to finally achieve heightened intimacy and interpersonal growth. The Gottman theory is grounded in the theory of emotionally focused couples therapy. Gottman is indebted to Johnson, who was the pioneer of clinical insights about emotional security. Johnson was the developer of emotionally focused couples therapy (EFCT). Gottman, based on Johnson’s point of view, claims that affect is not the problem, but it is central for understanding, compassion and changes, so Gottman suggests that couple therapists need to become an expert on emotion and help couples establish an emotional connection. Gottman illustrated his theory in seven stages and called it the Sound Relationship House Theory (SRHT). Gottman developed this theory to show how a relationship can have a good function or fail [2,5,6,33,65,66,70,80,81]. He offers a method for positive changes in relationships through psychoeducational, preventive and therapeutic interventions. 

#### 1.3.3. Reasoning for Selection of Communication Theory and Gottman’s Theory, the Alignment of Theories

Different couple interventions have shown ratios in improving marital satisfaction for unsatisfied couples in terms of the decrease in the demand–withdrawal communication pattern and encouraging them to use constructive communication [2,24,32,33,34,35,36,37,38]. Communication is the core concept of many family approaches and in Gottman’s theory, one of the more important concepts is focused on toxic communication patterns, described as “the four horsemen of the apocalypse”, which are criticism, defensiveness, contempt and stonewalling. Gottman’s theory utilized the concept of communication patterns to predict divorce and described them as “harsh start-ups”, “flooding”, “body language”, “failed repair attempts” and “bad memories” [2,65].

This study selected the Gottman approach (SRHT) for many reasons. The first reason is the depth and breadth of his research, which has established him as a significant player and influence in the field. Gottman is one of the most prolific researchers in marriage and family therapy [81,82]. The Gottman couples therapy method is an organized, goal-oriented and scientifically based therapy. Intervention strategies are based on empirical data from Gottman’s three decades of research involving more than 3000 couples [5].

What must be known is whether there are precise trajectories toward marital dissolution or marital stability that are systematically connected to the qualities of a marriage. This knowledge must come from prospective longitudinal studies, rather than from retrospective accounts of failed marriages. In this way, Gottman’s studies are a very good selection to look into the modification of marital communication as he has studied many couples for a long period of time with follow-ups to assess the effects of therapy.

The second reason is his theoretical framework and the extent of his programs. The Gottman approach is pragmatic and evaluates all behavioral, cognitive and emotional aspects. Gottman’s process theory of a cascade toward marital dissatisfaction and fibrinolysis incorporates both behavioral and social exchange theories [80], and his theoretical framework in the study of marital communications is systems theory, which can provide a whole explanation of the family structure.

The third reason for choosing Gottman has to do with one area in which he stands out among marital researchers: his concern with divorce. Unlike many researchers, Gottman has been particularly interested in divorce. Gottman is one marital researcher who constantly addressed divorce [48,70]. Gottman’s team can even predict which newlywed couples will get a divorce from the way they interact in just the first three minutes of a discussion. Among other things, they examine their heart rates, facial expressions and how they talk about their relationship to each other and to other people.

The fourth reason is that to be critical in the field of marital issues, it is important to consider analyses of the work of one who is both an integrated member and a critic of the field himself, and to judge just how far his criticisms go. Gottman is one of the few who tackles the question of divorce so explicitly, so it seems likely that he will be less inclined to make assumptions that inhibit the deep discussion of divorce in the larger discipline.

## 2. Method

This study generally hypothesizes that if participants empirically receive, using the Zoom platform, the online Gottman’s psychoeducational intervention (O-GPI), as the independent variable, their marital communication patterns (MCP), as the dependent variable, will be changed. Therefore, a single-blind parallel group, randomized controlled trials [83] with an experimental longitudinal design was chosen. Participants were observed four times at almost 13-month intervals (10 March 2019–2 May 2020). Therefore, in this study, the “long-lasting effect” of Gottman’s intervention was considered.

### 2.1. Screening and Selection Procedure

**Figure 1:**
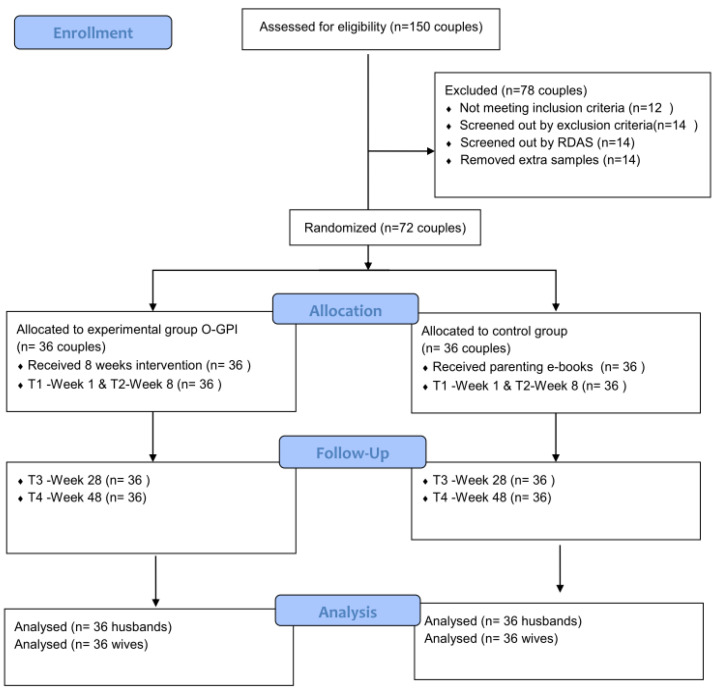
CONSORT flowchart.

Selecting the target sample was based on the stages shown in Figure 1: in the first stage, from 10 until 24 April 2019, for two weeks, posters promoting the Gottman psychoeducational programs were advertised in public newspapers and traffic-heavy websites, as well as giant billboards across highways in Shiraz, which is the sixth-largest metropolitan city in Iran. Shiraz is divided into 10 regional municipalities, and every region has at least five main public places. The advertisements distributed in these places had brief information on the main targets and advantages of the program to encourage heterosexual couples to join to enhance their marital communication. The advertisements invited couples to register for the program on the website of the family counseling center of Shiraz Medical Science University. One hundred and fifty couples signed up.

In the second stage, these couples were interviewed online via the Zoom platform by the first author and 138 of 150 couples were accepted based on the inclusion criteria (couples between 25 and 45 years old in a heterosexual marriage of between 1 and 7 years and who have stayed in Shiraz together for at least 2 years prior to the study). A total of 14 of 138 couples were excluded based on the exclusion criteria (5 wives in the short interview reported some severe domestic violence signs in their relationship, 4 couples were undergoing psychological treatment, 2 were polygamous and 3 husbands were addicts). Finally, the rest of the participants (*n* = 124 couples) were screened with the Revised Dyadic Adjustment Scale (RDAS) to remove couples with severe or without marital problems. Screening with RDAS prepared a sample of those couples (*n* = 86 couples) that were homogenous, with not much of a difference in terms of marital problems with the other couples. Therefore, the normal couples without severe conflict and marital maladjustment might achieve more benefit from Gottman’s psychoeducational intervention.

In the third stage, the sample size was determined based on the following factors: Cohen’s rule [84], the size effect of the repeated measure ANOVA, the within-between interactions, which was 0.15, and the level of significance, which was 0.05. Therefore, the effect size in this research was 0.15 with a power equal to 0.80. Additionally, in order to avoid statistical mortality, the initial sample size included a 15% dropout rate for each group. Hence, based on the above criteria, 72 couples were the appropriate sample size. Finally, the 72 couples that were eligible were selected from 86 couples through a simple random sampling method by using a random number table. Following that, eligible participants were assigned randomly into control and experimental groups based on the list of odd and even numbers.

At the pretest phase, T1, every couple in the control and experimental group received one envelope that includes two similar sets, one of which was considered for the wife and the other one for the husband. The two sets for one couple had the same code and comprised a consent form and instruments. Each pair of couples throughout all phases was identified with the same code that was only to be specified to the first author, and they were requested to remember this code until the study is completed at the end of the final phase. Participants were requested to fill the form and questionnaires and after completion, the surveys were returned via the researcher’s email.

At the intervention phase, participants (36 couples, *n* = 72 Individuals) in the experimental group attended eight consecutive intervention sessions via the Zoom platform (one session per week) from the first of May till the first of July 2019. They were requested to attend all sessions regularly with their spouse and fill out the consent letter before attending the online sessions. The experimental group was trained by the first author, who was qualified for the first level as a Gottman family trainer by the Gottman institute in 2011 in Singapore. Couples in the experimental group were expected to learn the appropriate skills concerning friendship, fondness, conflict management and the creation of common sense and values. The control group, meanwhile, did not attend any Gottman’s intervention sessions and was selected for comparison with the experimental group to show that the research outcomes are based on the effect of intervention and no other variables. Regarding ethical issues, the participants in the control group were offered e-books via email in the field of improving parental skills during the treatment duration. At the posttest and follow-up tests, all the recruited participants (in both experimental and control groups) were re-examined online with identical questionnaires at the posttest (T2, on the first of July 2019) and follow-up tests (T3, five months after posttest, on the first of December 2019 and T4, ten months after posttest, on the first of May 2020).

### 2.2. Data Collection Tools

The instruments for data collection were the Demographic Questionnaire, Revised Dyadic Adjustment Scale (RDAS) [74] and Communication Patterns Questionnaire (CPQ) [32]. In this study, the Persian version using the double-back translation method with appropriate psychometric procedures of RDAS [54] and CPQ [85] were conducted.

The 14-item RDAS is a revised version of DAS, which has a Likert scale with 32 items that are distributed among 4 sub-scales for measuring a couple’s relationship adjustment, whether they are distressed or non-distressed couples. CPQ is a questionnaire with 35 items under 6 sub-scales that evaluates individuals’ perceptions of the dyadic patterns of problem-solving behavior occurring in their couple’s relationship. In this study, only constructive communication (CC), total demand–withdraw communication (TWDC) and mutual avoidance communication (MAC) were evaluated in the 6 sub-scales of CPQ.

### 2.3. Description of the O-GPI Protocol

The protocol of the online Gottman’s psychoeducational intervention (O-GPI) was based on the Sound Relationship House Theory (SRHT) by Gottman [2,5,6,33,65,66,70,80,81,82], which was postulated to affect marital communication patterns through four main foundations, namely, “Relationship’s Friendship”, “Positive Perspective”, “Regulate Conflicts” and “ Create the Meaning System” [2,5,6,33,65,66,70,80,81,82].

This protocol followed the seven parts of SRHT to build a fundamental process for preventing destructive communication and dissolution [65], which is listed below [5,66]:

1. Build love maps;
2. Share fondness and admiration;
3. Turn toward each other;
4. Positive perspective;
5. Manage conflict;
6. Make life dreams come true;
7. Create shared meaning.

Based on the above, the first author was responsible in all eight sessions (each session lasted one and half hours for eight consecutive weekends) to describe the relevant information, strategies, therapeutic questions, review of previous session’s findings, consider the group feedback and participants’ exercise/homework between each session.

### 2.4. Statistical Data Analysis

The collected data from the above instruments were coded into the Statistical Package for the Social science (SPSS) version 22. A repeated measures one-way analysis of variance (RM-ANOVA) was performed to test the hypotheses and data analysis. The level of significance was set, a priori, at 0.05. The tests were carried out over four time points among 144 spouses (72 couples).

## 3. Result

Based on the selected demographic variables, approximately half of the participants were between 20 and 30 years old (46.5%), with a mean age of 32.25 years (SD = 5.96). More than 60% of participants were undergraduates and 69.4% of the participants reported that they had been married between 1 and 4 years (SD = 1.97). Of the study population, 39 couples had no children. Additionally, as expected, based on the chi-squared test and t-test results, there was no significant difference between the males’ and females’ demographic variables, and there were also no significant differences between the intervention and control groups in terms of their selected demographic variables. No significant differences were found between males and females in their CC, TWDC and MAC in the control and experimental groups. They were assigned randomly, and the data were independent observations. Additionally, the inferential statistics results of the factorial RM-ANOVA for the mean CC, TWDC and MAC scores between participants and the interaction between genders, groups and times are shown in Table 1 and Table 2 below.

### 3.1. Constructive Communication

**Figure 2:**
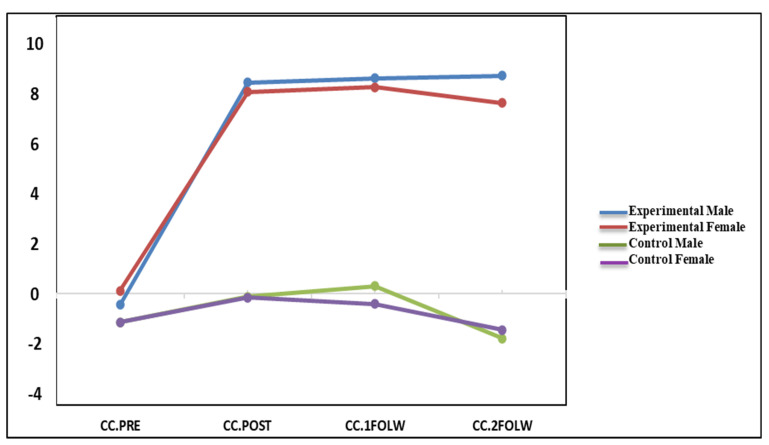
The mean for constructive communication in the experimental and control groups and between males and females across time.

The results in Table 1 have indicated that the mean CC scores for males and females in the experimental group were significantly higher than those for males and females in the control group, across the posttest, first and second follow-up tests. The partial eta-squared value for males (females) in the experimental group at T2 was 0.17 (0.14), which yielded a large effect of f = 0.45 (0.40). Therefore, based on Cohen’s (1977) guidelines for effect size, the findings showed that the main effect of O-GPI on the experimental group was higher than on the control group; this effect can persist over time and has a relatively long-lasting effect (at least for one year) for Iranian couples (See Figure 2). The results showed that 45% of the variability in the constructive communication among men and 40% among women might be due to O-GPI. There was no interaction gender effect in the revealed result, as males and females improved almost similarly in constructive communication.

### 3.2. Demand–Withdraw Communication

**Figure 3:**
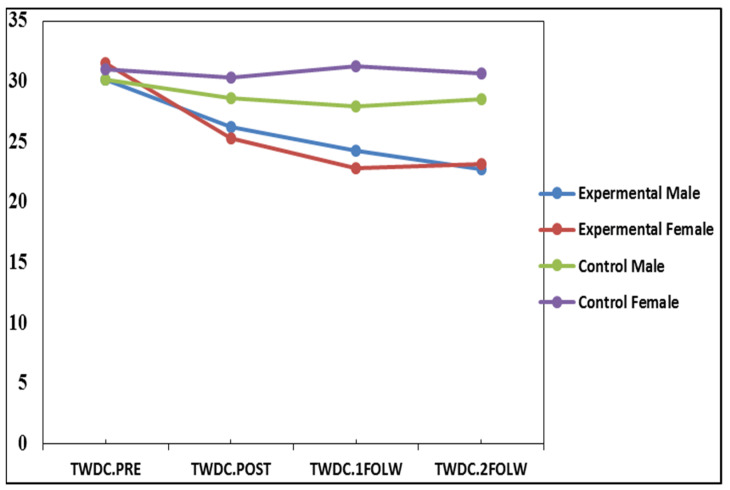
The mean of the total demand–withdraw communication in the experimental and control groups and between males and females across time.

In the current study, the results of the factorial RM-ANOVA for the mean TWDC scores between the experimental and control groups were significant for females across the posttest, first and second follow-up tests (*p* < 0.01); while they were not significant for males (Table 1). As shown in Figure 3, the findings demonstrated that the effect of O-GPI can persist over time and has a relatively long-lasting effect (at least one year) among Iranian couples. The partial eta-squared value for males in the experimental group was 0.21, which yielded a very large effect of f = 0.51. The partial eta-squared value for females was 0.30, which also yielded a very large effect of f = 0.65 using the guidelines proposed by Cohen [84]. The results showed that 51% in the variability in the demand–withdraw communication among men and 56% among women might be due to O-GPI. Interestingly, there was a significant interaction gender effect in the revealed result; males and females did not improve similarly in demand–withdraw communication.

### 3.3. Mutual Avoidance Communication

**Figure 4:**
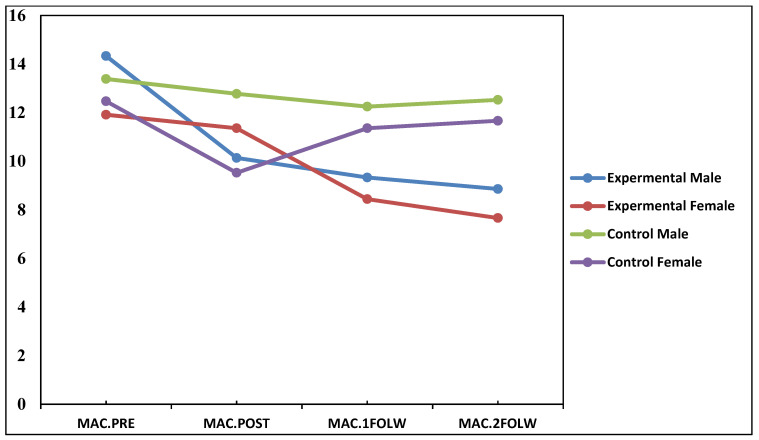
The means of mutual avoidance communication in the experimental and control groups and between male and female across time.

In this study, it was hypothesized that the online Gottman’s psychoeducational intervention can decrease mutual avoidance communication. As can be seen in Table 1, the results between the experimental and control groups were significant for MAC among males and females across the posttest, first and second follow-up tests (*p* < 0.01). The mean scores show that the mutual avoidance communication pattern for males and females in the experimental group are lower than those in the control group across the period of the study. Therefore, the findings revealed that the effect of the O-GPI could persist over time and for a relatively long time (at least for one year) for Iranian couples. The partial eta-squared value for males in the experimental group was 0.27, which provided a very large effect f = 0.60; for females, it was 0.28, which provided a very large effect of f = 0.62. As shown in Figure 4, the results show that 60% of the variability in mutual avoidance communication among males and 62% in females could be due to Gottman’s psychoeducational intervention. Hence, the findings indicated that Gottman’s psychoeducational intervention has a very large effect in decreasing the mutual avoidance communication pattern among Iranian couples.

In this study, the interactions between groups, gender and times for all variables (CC, TWDC and MAC) were interesting. The results of the Bonferroni test in Table 2 indicated that there are no significant interaction effects over time as a function of gender or group. The main effect of gender and interaction between gender and group was not significant. In other words, both males and females exhibited the same pattern of CC, TWDC and MAC over time; so, both genders were affected equally by the standard effect of the intervention on their communication.

## 4. Discussion

The current study attempts to draw on the theoretical framework and relevant research to show the vital effect of communication patterns among couples [26,27,86]. Improving constructive communication patterns (CC) as one of the marital communication patterns is very critical for increasing marital satisfaction [30,31,87], marital quality [28,88] and preventing a decrease in mental health. The results are parallel to Gottman’s findings. Gottman emphasizes in the training how enhancing friendship with the building of a love map, an admiration system and enhancing emotional connection through turning towards one another can constructively improve a couple’s communication [24,42,46,70,82,89].

Regarding the significant similarities between this study and previous research, it was again proven that the differences between the control and experimental groups are based on their attendance of the intervention. It is debatable that concepts of Gottman’s intervention are easy for localization because the results indicated a large effect size of the intervention to improve a couple’s constructive communication. Both clients and family counselors can utilize the familiar concepts of Gottman’s intervention since there are no significant challenges between Iranian culture and Gottman’s concepts [24]. Additionally, other results showed that the differences between before and after the intervention were significant. Therefore, it can be concluded that the effect of the intervention may last up to one year. These findings are supported by prior studies [21,24,32,43,70,90] that have noted the importance of maintaining the effect of the Gottman therapy on marital communication. As studies have indicated, Gottman’s intervention can be effective because of the low relapse rate; interestingly, it was concluded that the effect of the Gottman intervention in this research will be maintained, and couples can utilize all the acquired skills in their real-life situations without any significant relapse [2,5,6,33,65,66,70,80,81].

On the other hand, many studies have highlighted that the demand–withdraw communication pattern is a negative communication pattern [28] because one spouse attempts to communicate by demanding or nagging, or even with contempt, while the other partner avoids talking and solving the problem. This pattern is related to the longitudinal decays in marital satisfaction [28,91]. Couples are trapped with the triggered stress and strain, which will lead to more conflict. It is necessary to deal with it, otherwise it will be easy for the negative emotions to escalate [92]. Regarding Christensen’s study, [7] as well as Gottman’s [5], whenever each partner fails to accept the other partner’s influence and their emotions, the communication pattern will be emotional withdrawal, and it will be illustrated either by males or females. As a result, they will be dissatisfied with their interactions [5,76,81]. The findings of our study are in line with previous study findings [65,66,78,93], which have demonstrated their efficacy in increasing marital satisfaction for distressed couples. The findings of this study are parallel to those of Gottman [6], who introduced a type of ailing couple that is called the conflict-avoidant style. These couples are equal to the demand–withdraw couple type introduced by Christensen. Gottman showed that after an intervention, this type of couple could change their ailing communication pattern. The results showed that the differences between before and after the intervention were significant. In other words, the effect of the intervention may last up to one year. Hence, the couple can utilize and adjust their acquired skills from the Gottman psychoeducational intervention. Based on this approach, couples can maintain the intervention effect in real life. Therefore, it is possible to observe the patterns of “Master” and “Disaster” couples in their real relationships. When they utilize these skills, they can become a master in decreasing the negative effect of the demand–withdraw communication pattern. If they fail to use these skills, there will be disastrous consequences, and thus lower marital satisfaction. One interesting finding of this study shows that both males and females improved with large effect sizes, but they do not improve similarly. These results are encouraging, particularly since the intervention is relatively inexpensive compared with the cost of carrying out a typical marital therapy outcome study. In short, the findings indicated that O-GPI is effective in decreasing the pattern of demand–withdraw communication among Iranian couples.

In the pattern of mutual avoidance, the absence of a negative interaction can confuse the couple therapists, and also the couple may think that there is no problem in the relationship. However, Gottman [2] illustrated that this situation is full of risk because of emotional disengagement. These couples do not make any emotional connection, and there is almost no humor or affection with each other in terms of emotional issues; thus, it can be said that their relationship is dead. Furthermore, pandemic-triggered stress can increase the influence of contextual vulnerabilities and, hence, couples that are already facing difficulties to achieve basic needs may have restricted cognitive, emotional and social support in handling the additional stress. They realize that they are in an unsafe situation, and experience high levels of stress and anxiety [62], which is related to deconstructive relationships.

It is expected that after the intervention the couples could be able to turn toward each other instead of turning away, and they will feel that their partners are responsive to their needs, take their partner’s perspective in to account and engage constructively to solve problems [82]. The result is corroborated by the findings of a great deal of previous work in the field of communication and literature on the effectiveness of the Gottman therapy [2,5,6,33,65,66,70,80]. Furthermore, the present findings seem to be consistent with other research that found that there are no gender biases in Gottman’s intervention, and both males and females can utilize the intervention’s advantages [82].

### 4.1. Implications of Results

Without a doubt, the social and daily family lives in many countries, including Iran, have been changed by many stressful and triggering events. As a result of the social and family stress, families struggle with the negative effects of stress. Families have experienced many challenges based on the confinement-related stress, social disruption, emotional distress, fear and panic due to high risk of illness, death and loss in all stressful occurrences. To reduce these negative effects on both individual well-being and intimate and family relationships, improving marital communication through an online Gottman psychoeducational intervention is more practical. It is worth noting that extracting concepts from Gottman’s theory can be of practical help to couple therapists, family counselors and psychologists and other professionals for three reasons. First, they can help couples deal with the challenges of family problems and crises. Second, they will find this method is very applicable and has familiar concepts for their clients, reducing the tendency among some clients to refuse therapy. Third, this study found that by utilizing questionnaires provided in Gottman’s theory, the therapist could evoke significant information from couple interactions. If therapists take sufficient time to assess their clients’ interactions carefully, they can understand the relationship’s strengths and determine which areas need development and enhancement before beginning treatment.

Additionally, this study has practical implications for clients who attend an intervention. This intervention can act as a self-monitoring strategy, so couples can utilize the strategy and concepts by themselves and thus need not depend on therapists step by step. The results will be implied for them at all times, particularly when they are under stressful events and significant social and economic disruption, given this knowledge of how to show their love and respect to the others and how they can manage their conflict with dialogue instead of destructive communication patterns, such as demand–withdrawal or mutual avoidance communication. Couples who acquired skills from an online Gottman psychoeducational intervention will be vaccinated from unsafe feelings, moving away from fostering high levels of stress and anxiety that they normally experience in stressful events. As other studies have shown [46,93], the Gottman intervention is easily adjusted from clinical and educational settings to real settings, such as normal individual and marital lives of spouses. The results of this study improve the knowledge of couples training in Iran and the important role that couple’s intervention plays. This important issue can encourage the attendance of Iranian couples, as well as encourage professionals to conduct online Gottman psychoeducational intervention.

### 4.2. Limitations

Although this study enhances knowledge of the effect of O-GPI on marital communication patterns, it faces three main limitations:

1. Sample characteristics. The generalizability of this study is limited only to Iranian heterosexual couples residing in Shiraz.
2. Methodology. The experimental design, randomization and minimization of individual differences were conducted; however, it was impossible to rule out all confounding variables, such as their motivation and cooperation rates, particularly in online sessions. Additionally, it is highly likely that participants had other information sources, such as friends, the Internet, TV and books, which could be used to improve their marital quality. This is not something that the researcher could control.
3. Measurement tools. Though participants were reassured that their responses would be protected and anonymized by the researcher, it is probable that some participants answered questionnaires with low confidence or for achieving more social acceptance. It is also possible that the accurate meaning of the questions was misunderstood and some couples avoided seeking clarification for each question.

## 5. Conclusions

Although to date various methods have been developed and introduced to improve marital quality and especially marital communication, only a few rare marital interventions are conducted via online platforms. The current study for understanding the effect of the eight online interventions using Gottman couples therapy compared the function of the experimental group to control participants. The results indicated that this intervention has long-lasting effects in real life among Iranian couples after a one-year follow-up. Additionally, the results emphasized the free gender-direction effect and have an equal effect on both males and females. Hence, this study concluded that the online Gottman’s psychoeducational intervention is an effective intervention with standard effects without gender approaches to improve marital communication among 72 Iranian couples. The results suggested that couples who participated in Gottman’s intervention had significantly greater enhancements in their constructive communication than those who did not. Additionally, they meaningfully decreased their demand–withdraw communication and mutual avoidance. These comparisons between both experimental and control group functions were conducted at the pretest, posttest, and the first and second follow-up tests. One positive aspect of this study was to encourage both spouses, especially husbands, to regularly attend all intervention sessions. Regular attendance and commitment to be active in sessions and attend each of the four evaluation tests (pretest, posttest, first and second follow-ups) was a very positive indicator, given the recognized difficulty and low motivation to attend therapy sessions, especially for Iranian men.

## Figures and Tables

**Table 1:** Pairwise comparison between the control and experimental groups for both male and female across time.

				CC				TWDC				MAC			
Time	Gend	Group		MD	SE	*p*	η^2^	MD	SE	*p*	η^2^	MD	SE	*p*	η^2^
Pre test	Male	Ex	Con	0.64	2.22	0.77	0.01	−0.03	1.39	0.98	0	0.94	1.08	0.38	0.05
	Fem	Ex	Con	1.14	2.22	0.61	0.02	0.53	1.39	0.70	0.01	−0.56	1.08	0.61	0.02
Post test	Male	Ex	Con	7.69	1.75	<0.01	0.17	−2.36	1.41	0.01	0.21	−2.64	0.92	0.05	0.27
	Fem	Ex	Con	7.39	1.75	<0.01	0.14	−5.06	1.41	<0.01	0.30	1.83	0.92	0.05	0.28
FU 1	Male	Ex	Con	7.47	1.62	<0.01	0.13	−3.69	1.58	0.01	0.04	−2.92	0.89	0.001	0.07
	Fem	Ex	Con	7.78	1.62	<0.01	0.14	−8.36	1.58	<0.01	0.17	−2.92	0.89	0.001	0.07
FU 2	Male	Ex	Con	9.44	1.73	<0.01	0.18	−5.75	1.72	0.01	0.07	−3.67	0.86	<0.01	0.11
	Fem	Ex	Con	8.17	1.73	<0.01	0.14	−7.50	1.72	<0.01	0.12	−4.00	0.86	<0.01	0.13

CC = Constructive Communication, TWDC = Total Withdraw/Demand Communication, MAC = Mutual Avoidance Communication; SE = Stander error, η^2^ = Effect size.

**Table 2:** The interactions between times, groups and genders.

				CC				TWDC				MAC			
Time	Gen	Group		Mean Differences	SE	*p* value	η^2^	Mean Differences	SE	*p* value	η^2^	Mean Differences	SE	*p* value	η^2^
Pre test	Male	Ex	Control	0.64	2.22	0.77	0.01	−0.03	1.39	0.98	0	0.94	1.08	0.38	0.05
	Fem	Ex.	Control	1.14	2.22	0.61	0.02	0.53	1.39	0.70	0.01	−0.56	1.08	0.61	0.02
Post test	Male	Ex.	Control	7.69	1.74	<0.01	0.16	−2.36	1.41	0.09	0.20	−2.64	0.92	0.05	0.27
	Fem	Exp.	Control	7.39	1.77	<0.01	0.14	−5.06	1.41	<0.01	0.30	1.83	0.92	0.05	0.27
FU 1	Male	Exp.	Control	7.47	1.62	<0.01	0.13	−3.69	1.58	0.02	0.04	−2.92	0.89	0.01	0.07
	Fem	Exp.	Control	7.78	1.62	<0.01	0.14	−8.36	1.58	<0.01	0.17	−2.92	0.89	0.01	0.07
FU 2	Male	Exp.	Control	9.44	1.73	<0.01	0.18	−5.75	1.72	0.01	0.07	−3.67	0.86	<0.01	0.11
	Fem	Exp.	Control	8.17	1.73	<0.01	0.14	−7.50	1.7	<0.01	0.12	−4.00	0.86	<0.01	0.13

CC = Constructive Communication, TWDC = Total Withdraw/Demand Communication, MAC = Mutual Avoidance Communication SE = Stander error, η^2^ = Effect size.
